# AAV-mediated MUC5AC siRNA delivery to prevent mucociliary dysfunction in asthma

**DOI:** 10.1038/s41434-025-00564-3

**Published:** 2025-08-23

**Authors:** Sahana Kumar, Maria Corkran, Yahya Cheema, Margaret A. Scull, Gregg A. Duncan

**Affiliations:** 1 Fischell Department of Bioengineering, University of Maryland, College Park, MD USA; 2 Department of Cell Biology & Molecular Genetics, Maryland Pathogen Research Institute (MPRI) University of Maryland, College Park, MD USA

**Keywords:** Respiratory tract diseases, Gene therapy

## Abstract

The main structural components of mucus produced in the lung are mucin 5B (MUC5B) and mucin 5AC (MUC5AC) where a relatively higher expression of MUC5B is typical in health. In the lungs of individuals with asthma, there is a shift from MUC5B to MUC5AC as the predominantly secreted mucin which has been shown to impair mucociliary clearance (MCC) and increase airway mucus plug formation. Given its role in asthmatic lung disease, MUC5AC represents a potential therapeutic target where a gene delivery approach could be leveraged to modulate its expression. For these purposes, we explored adeno-associated virus serotype 6 (AAV6), as a viral gene vector to transduce airway epithelial cells and reduce MUC5AC expression via siRNA delivery. We confirmed that AAV6 was able to transduce epithelial cells in vitro as well as in the airways of healthy mice in vivo with high transgene expression in mucus-secreting goblet cells. Using multiple particle tracking analysis, we observed that AAV6 was capable of penetrating both normal and MUC5AC-enriched mucus barriers. AAV6 carrying MUC5AC-targeting siRNA was evaluated as a prophylactic treatment in HAE cell cultures before IL-13 challenge. IL-13 stimulated HAE cultures treated with AAV6-MUC5AC siRNA had significantly reduced MUC5AC mRNA and protein expression compared to untreated controls. Mucociliary transport in IL-13 stimulated HAE cultures was also maintained and comparable to healthy controls following AAV6-MUC5AC siRNA treatment. Together, these findings support that AAV6 may be used as an inhaled gene therapy to suppress MUC5AC overexpression and restore normal airway clearance function in asthma.

## Introduction

Asthma is a chronic lung disease, affecting millions worldwide, adults and children alike [1]. Individuals with asthma typically experience coughing, difficulty breathing and wheezing which impacts their day to day activities and overall quality of life [2, 3]. Allergic asthma is associated with airway inflammation, airway remodeling, and impaired clearance of mucus from the airway by coordinated beating of cilia on the epithelium, a process known as mucociliary clearance (MCC) [4, 5]. As asthma worsens, excess mucus is produced leading to mucus plug formations due to impaired MCC [6]. Standard treatments for asthma, including corticosteroids and bronchoconstrictors, are focused primarily on managing the symptoms of inflammation and airway constriction. However, patients endure the disease for years and long-term use of these drugs can cause significant financial burden and have off-target effects [7–9]. Beyond over-the-counter expectorants which may provide temporary symptom relief, there has been minimal progress in therapeutic development to address muco-obstruction and airway plugging observed in severe asthma.

To achieve long-lasting therapeutic effects, gene therapy strategies have been developed and evaluated for their ability to inhibit or reduce specific Th2 inflammatory pathways in asthmatic airways. In addition, local delivery of gene therapies for asthma via inhaled or intranasal administration can enable less frequent dosing and reduce off-target effects [10]. So far, targeted gene therapeutic approaches for asthma have used both viral and non-viral vectors, loaded with either antisense oligonucleotides, siRNA, or mRNA that aim to antagonize or silence inflammatory mediators or shift the immune response from a Th2 response to a Th1 response [11–16]. In previous work, an adeno associated virus (AAV) encoding for receptor antagonist of IL-4RA was able to alleviate the Th2 allergic response in a mouse model of allergic asthma. This strategy also reduced mucus hypersecretion and airway hyperresponsiveness in ovalbumin sensitized mice [11]. Another study used AAV for sustained expression of IL-12 to enhance Th1 responses in OVA sensitized asthmatic mice where they observed reduced Th2 cytokines in treated mice [17].

While inflammation and immune mediators are an important therapeutic target, fatal asthma attacks are often caused by mucus plugs which can partially or fully occlude small and large airways leading to restricted airflow [18–20]. Within the lung, mucus is formed by gel-forming mucin glycoproteins, mucin 5B (MUC5B) and mucin 5AC (MUC5AC) where each of these mucins possess distinct biochemical (e.g., glycosylation pattern) and biophysical properties (e.g., macromolecular assembly, crosslinking) [6]. Healthy airway mucus is comprised predominantly of MUC5B and with relatively lower levels of MUC5AC, while the contrary is observed in asthmatic airways, with MUC5AC being the predominant mucin [21]. Prior studies have shown that this change in mucus composition contributes to viscous mucus, impaired MCC, and mucus plug formations in airways [18, 22, 23]. These prior studies also established IL-13 mediated goblet cell hyperplasia in asthma leads to MUC5AC hypersecretion and pathological mucus production in asthma. A prior study targeted microRNA miR-141, which regulates IL-13-induced mucus secretion, and showed miR-141 disruption reduced goblet cell hyperplasia, MUC5AC expression, and total secreted mucus [24]. As such, MUC5AC warrants attention as a therapeutic target for treating asthma which could be addressed using a gene therapy strategy.

AAV is one of the leading viral vectors for gene therapy applications because it has broad tissue tropism and is non-pathogenic. Further, several AAV-based approaches have obtained FDA approval with many others in clinical trials [25, 26]. To deliver MUC5AC-siRNA, AAV serotype 6 (AAV6) was selected for these studies based on its ability to transduce airway epithelial cells in vitro and in vivo [27, 28]. We have also shown in prior work that AAV6 can penetrate hyper-concentrated mucus produced by individuals with cystic fibrosis lung disease and transduce airway epithelium in the βENaC transgenic mouse model of muco-obstructive lung disease [29]. Building on this prior work, we determined in the current work that AAV6 can transduce secretory cells in mouse airways and as such, is a relevant gene vector for MUC5AC siRNA delivery. To determine its effectiveness in the context of disease, we further evaluated if AAV6 could successfully penetrate pathological asthma mucus for delivery of MUC5AC siRNA and improve MCC in IL-13 stimulated human airway epithelial cultures. The results of this work could open new avenues for inhaled therapeutics for asthma and other muco-obstructive lung diseases.

## Results

### AAV6 transduction in mouse airway and lung epithelium

**Fig. 1:**
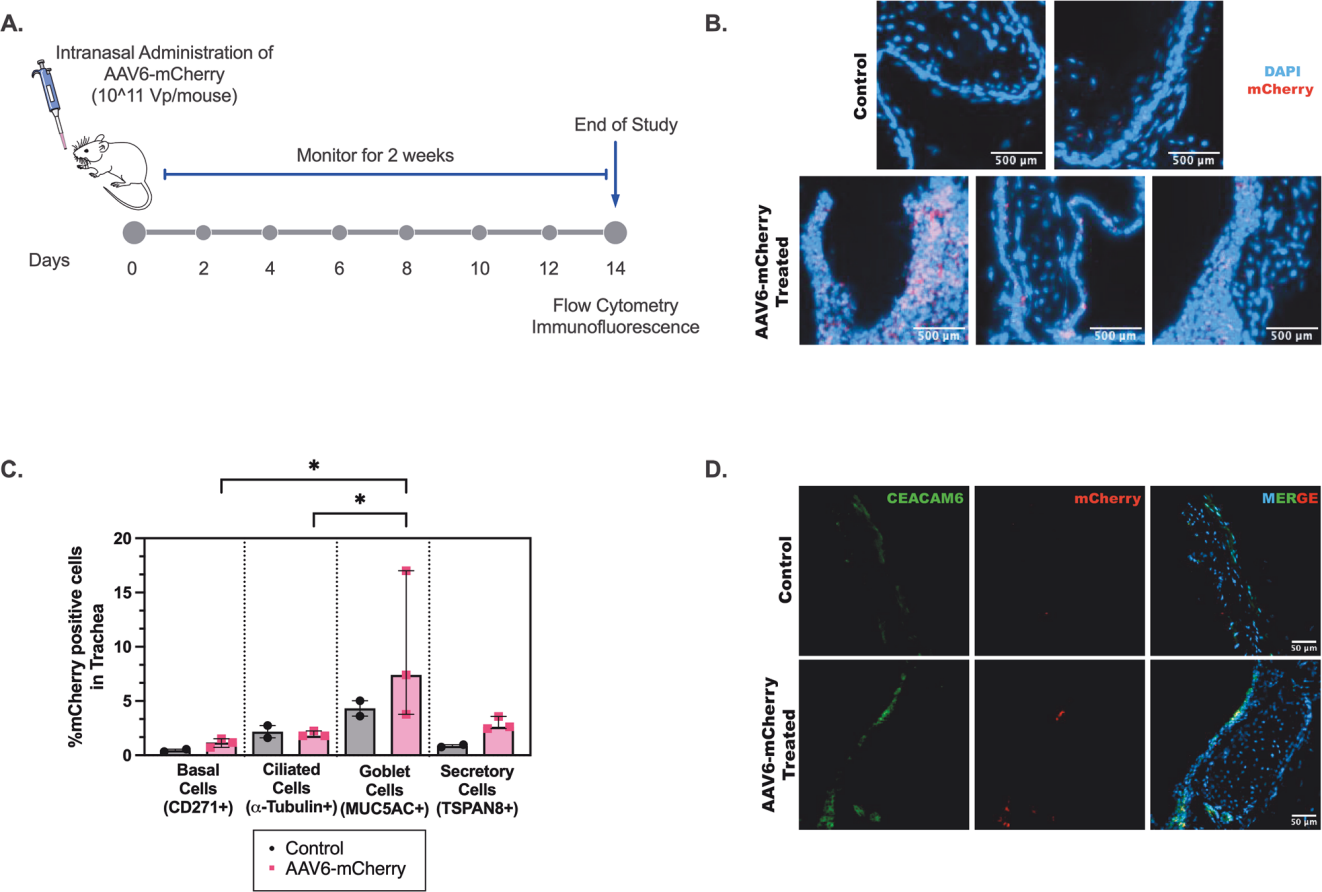
In vivo AAV6 transduction in mouse airway epithelial cells. **A** Schematic illustration showing intranasal AAV6 administration in BALB/c mice and overall study design. **B** Immunofluorescence images of trachea sections in control (PBS) and AAV6-mCherry infected mice. mCherry transduction is shown in pink. Scale bar = 500 µm. **C** Bar Graphs show % mCherry positive cells normalized to control in different airway epithelial cells from single cell suspension of excised trachea. *p < 0.05 by Ordinary one-way ANOVA. Each dot represents results from trachea tissue from one mouse. **D** Immunofluorescence images of trachea sections in control (PBS) and AAV6-mCherry infected mice with secretory cell marker Ceacam6 (green). mCherry and Ceacam6 colocalization is seen in yellow. Scale bar = 50 µm.

AAV6 has demonstrated efficient gene delivery to airway epithelial cells when administered intranasally or intratracheally in mice, consistent with its effective penetration and cellular uptake in the respiratory tract. However, we note systemic delivery does not result in lung-targeted transduction, and thus AAV6 is not inherently lung-tropic [28–32]. To examine and validate that AAV6 can deliver genes to airway epithelial subtypes, we conducted an in vivo biodistribution study (Fig. 1). Briefly, AAV6 encoding for an mCherry reporter was intranasally administered into 6-week-old BALB/c mice (5 males and 5 females) at a dose of 10^11^ viral particles/mouse in 15 µl PBS (Fig. 1A) and equal volume of PBS in controls. As expected, mice in both the control and treated groups did not show any signs of adverse effects with normal weight gain observed over the course of the study (Supplementary Fig. S1A). After 14 days, mice were euthanized in order to collect trachea and lung tissue to measure mCherry expression in epithelial subtypes by flow cytometry and immunofluorescence. Immunofluorescence analysis showed mCherry expression in tracheal sections indicating successful AAV6 transduction (Fig. 1B). Quantification of mCherry in tracheal epithelial subsets via flow cytometry indicated a significantly higher transduction in MUC5AC+ goblet cells subsets compared to basal and ciliated cells as well as increased transduction in TSPAN8+ secretory cells (Fig. 1C). To verify that mCherry transduced secretory cells, immunofluorescence analysis of tracheal sections expressing CEACAM6 secretory cell marker showed mCherry colocalization (Fig. 1D). Immunofluorescence in lung samples yielded less conclusive results with low mCherry expression in immunofluorescence images of lung sections (Supplementary Fig. S1B). Interestingly, there was significantly higher transduction in TSPAN8+ epithelial cell subtypes compared to other epithelial subtypes (Supplementary Fig. S1C). However, immunofluorescence analysis of lung sections showed reduced Ceacam6 expression and no colocalization was observed (Supplementary Fig. S1D).

### AAV6 is diffusive in asthma-like, MUC5AC-enriched mucus

**Fig. 2:**
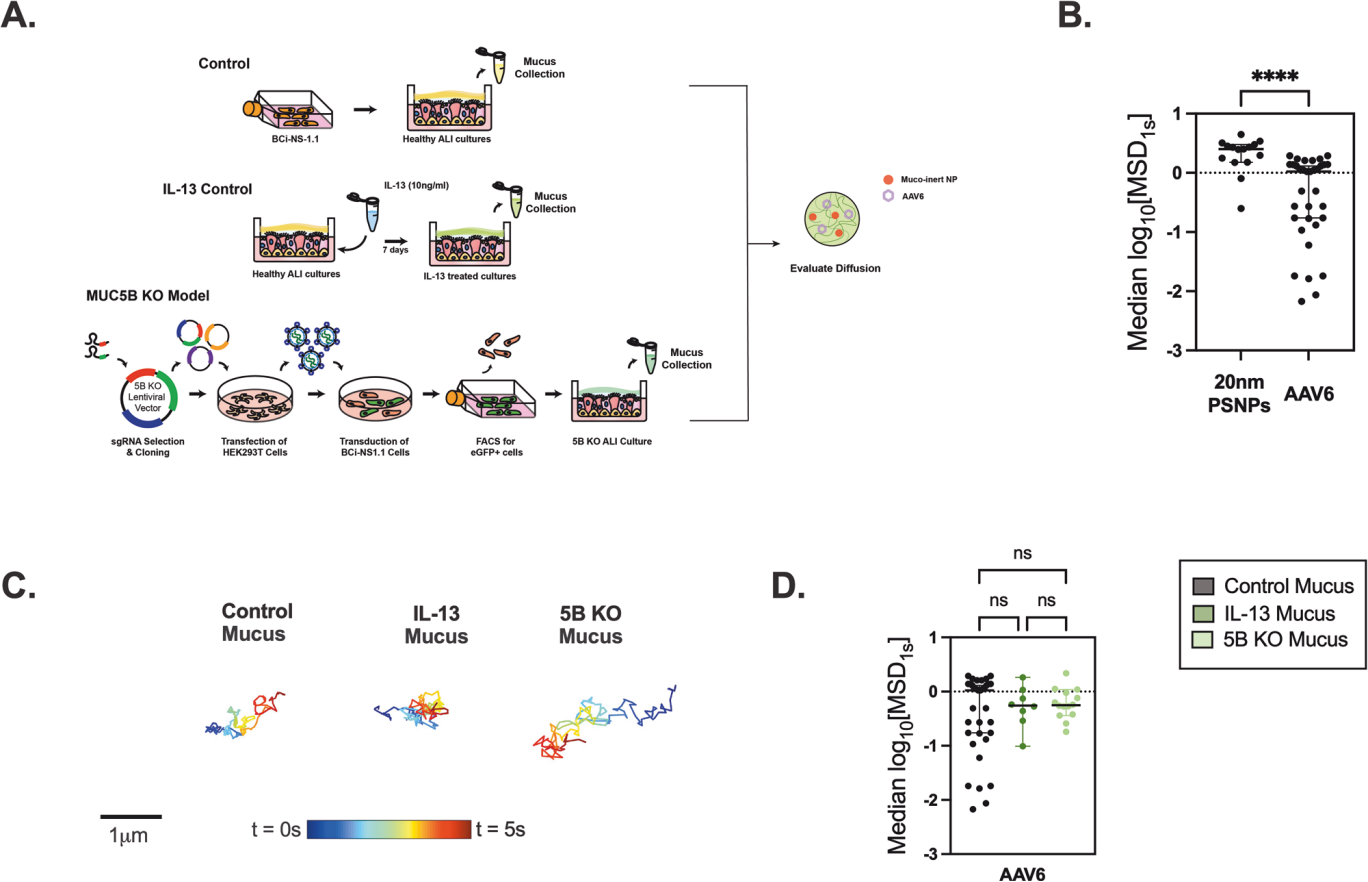
AAV6 diffusion in control and IL-13 treated mucus from in vitro cultures. **A** Schematic illustration showing the different in vitro models from which mucus was collected. BCi-NS1.1 cells were grown at ALI to establish Healthy ALI cultures. Healthy differentiated ALI cultures were stimulated with IL-13 (10 ng/ml) for 7 days to produce MUC5AC rich mucus. MUC5B KO cultures were generated using lentiviral mediated delivery of sgRNA and CRISPR-Cas9. Mucus from healthy controls, IL-13 treated cultures and MUC5B KO cultures were collected and AAV6 diffusion was measured in comparison to muco-inert nanoparticles of similar size. **B** Scatter plots of measured median log_10_[MSD_1s_] for AAV6 and 20 nm muco-inert nanoparticles in healthy mucus collected from ALI cultures. ****p < 0.0001 by Mann–Whitney Test. **C** Representative trajectories of AAV6 diffusion in control mucus, IL-13 treated mucus, and MUC5B KO mucus. Trajectory color changes with time with dark blue indicating 0 s and dark red indicating 5 s. Scale bar = 1 µm. **D** Scatter plots of measured median log_10_[MSD_1s_] for AAV6 in healthy control mucus, IL-13 treated mucus, and MUC5B KO mucus. *p* > 0.05 by Kruskal–Wallis test with Dunn’s correction. Each dot represents data from 1 video (*n* ≥ 3).

After confirming the relevance of AAV6 as a gene vector for pulmonary delivery to target mucus secreting cell types, we then characterized AAV6 diffusion in normal and MUC5AC-rich mucus produced in vitro from air liquid interface (ALI) cultures of human airway epithelial (HAE) cells to mimic mucus from asthmatic patients. Previous studies have shown that stimulation of HAE cultures with IL-13, which is a key cytokine in allergic asthma, causes goblet cell hyperplasia, increased MUC5AC production and reduced mucociliary transport [23, 33–35]. As such, we established this in vitro asthma model by stimulating BCi-NS1.1 HAE cultures grown at ALI with IL-13 (10 ng/ml) for 7 days as previously described [22, 34] (Fig. 2A). These cultures will be referred to as IL-13 treated HAE cultures. We also utilized a previously established model in BCi-NS1.1 cells where MUC5B gene expression is knocked-out via CRISPR-Cas9 leading to the production of MUC5AC-enriched airway mucus (Fig. 2A). [36] The diffusion of fluorescently labeled AAV6 (~20 nm in diameter) [37] and 20 nm diameter muco-inert nanoparticles (NP) was measured in control mucus. We confirmed that the muco-inert NPs were in the same size range as AAV6 by dynamic light scattering measurements. The diffusion rates for AAV6 and NPs were measured in the same regions of interest using fluorescence video microscopy. Based on the multiple particle tracking analysis and measured mean squared displacement at time scale of 1 s (MSD_1s_), the diffusion rate for muco-inert NPs were significantly higher than the rate for AAV6 (Fig. 2B). We compared AAV6 diffusion in regular (healthy) mucus, IL-13 mucus, and mucus from MUC5B knockout (MUC5B KO) cultures, which are predominantly composed of MUC5AC to see how this change in mucus composition would impact viral diffusion. We found AAV6 is diffusive and traverses through the gel network comparably in control mucus, IL-13 mucus, and MUC5B-KO mucus as shown by the trajectories (Fig. 2C) and median log_10_(MSD_1s_) values (Fig. 2D) with no significant difference between mucus types.

### AAV6 effectively transduces IL-13 stimulated airway epithelial cells in vitro

**Fig. 3:**
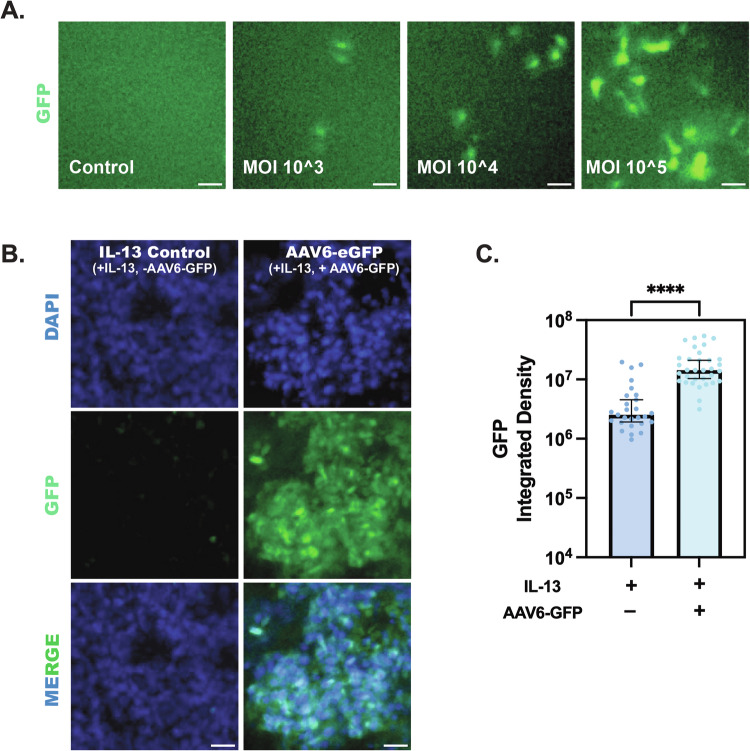
AAV6 transduction in healthy and IL-13 treated ALI cultures in vitro. **A** Representative images showing AAV6-eGFP transduction in undifferentiated BCi-NS1.1 cultures at MOI of 10^3^, 10^4^, and 10^5^. Scale bar = 50 µm. **B** Representative images showing AAV6-eGFP transduction in IL-13 treated ALI cultures with respect to untransduced IL-13 treated controls. Scale bar = 20 µm. **C** Bar graphs showing GFP integrated density quantified from immunofluorescence images (**B**) in IL-13 treated AAV6-eGFP transduced ALI cultures with respect to untransduced IL-13 treated controls (*n* = 9). ****p < 0.0001 by Welch’s *t* test. Each dot represents different images.

We next wanted to verify that AAV6 can transduce IL-13 stimulated HAE cultures in vitro. We first investigated the transduction efficiency of AAV6 expressing enhanced green fluorescence protein (eGFP) in undifferentiated BCi-NS1.1 lung basal cells at multiplicities of infection (MOI) of 10^3^, 10^4^ and 10^5^ viral particles per cell. AAV6 mediated GFP expression was evaluated 72 h post infection using fluorescence imaging. Successful transduction was observed in cultures inoculated at a MOI as low as 10^3^, with a substantial increase in fluorescence detection with MOI of 10^4^ and 10^5^ (Fig. 3A). Based on this, AAV6-eGFP transduction was evaluated at MOI of 10^5^ in IL-13 treated differentiated ALI cultures and GFP expression was evident 72-hours post transduction (Fig. 3B). Using fluorescent micrographs from multiple HAE cultures, GFP integrated density was measured in AAV6-eGFP transduced ALI cultures with respect to controls (Fig. 3C). There was a significantly higher GFP expression intensity in the transduced groups compared to controls.

### AAV6 mediated delivery of MUC5AC-siRNA in IL-13 stimulated human airway epithelial cells

**Fig. 4:**
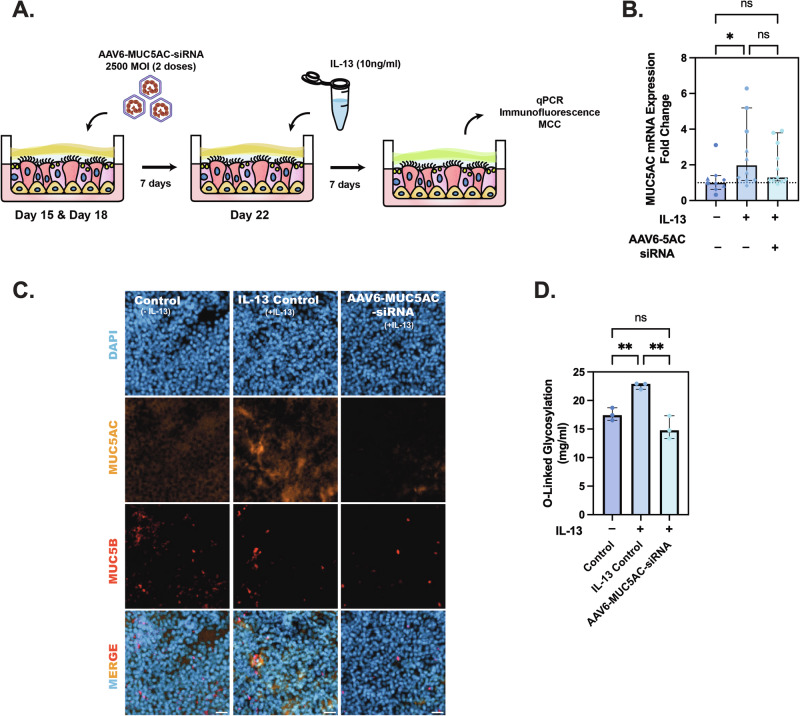
AAV6 delivers MUC5AC siRNA into IL-13 treated ALI cultures. **A** Schematic illustration demonstrating 2 dose pre-treatments with 2500 MOI of AAV6 expressing MUC5AC siRNA (AAV6-5AC siRNA) in ALI cultures before challenging with IL-13. **B** Scatter plot showing MUC5AC mRNA fold change in IL-13 treated control and AAV6-5AC siRNA treated groups with respect to untreated control. Shapes represent different experimental trials. *p < 0.05 by one-way ANOVA. **C** Representative images showing MUC5B and MUC5AC protein expression in untreated control, IL-13 treated control and AAV6-5AC siRNA treated groups. Scale bar = 25 µm. **D** Bar graphs showing O-Linked glycosylation in controls and treated groups. *p < 0.001 by Ordinary one-way ANOVA.

AAVs can be effectively packaged with siRNA, which are short 21-nucleotide dsRNA molecules, for gene therapy to target disease-causing genes [38]. To test the efficacy of AAV6 mediated silencing of MUC5AC expression, we transduced ALI cultures with 2 doses of AAV6 carrying MUC5AC siRNA (AAV6-5AC siRNA) on day 15 and day 18 of differentiation respectively, at an MOI of 2500 and allowed 72 h for transduction for each dose. On day 21, these cultures were challenged with IL-13 for 7 days. On day 28, the cultures were assessed for MUC5AC RNA and protein expression (Fig. 4A). We performed qPCR to measure MUC5AC mRNA levels and found that groups treated with AAV6-5AC siRNA had comparable MUC5AC mRNA levels to untreated control whereas the IL-13 treated control was ~1.5 times higher than the treated group and ~2 times higher than untreated control (Fig. 4B). To measure MUC5AC and MUC5B protein expression, we performed immunofluorescence staining in untreated, IL-13 treated control, and AAV6-5AC siRNA treated groups and quantified the integrated density of MUC5AC and MUC5B expression. There was increased MUC5AC expression in IL-13 treated controls compared to untreated control and AAV6-5AC siRNA treated groups (Fig. 4C & Supplementary Fig S2A) showing that the treated groups maintained baseline MUC5AC expression similar to healthy controls. There was also increased mucin content as reflected by the O-linked glycosylation quantification in IL-13 treated groups with lower and comparable mucin content in untreated control and AAV6-5AC siRNA treated group (Fig. 4D). MUC5B expression was comparable across all groups with no significant difference (Fig. 4C & Supplementary Fig. S2B).

### Impact of AAV6 mediated MUC5AC siRNA treatment on mucociliary transport in IL-13 stimulated human airway epithelial cell cultures

**Fig. 5:**
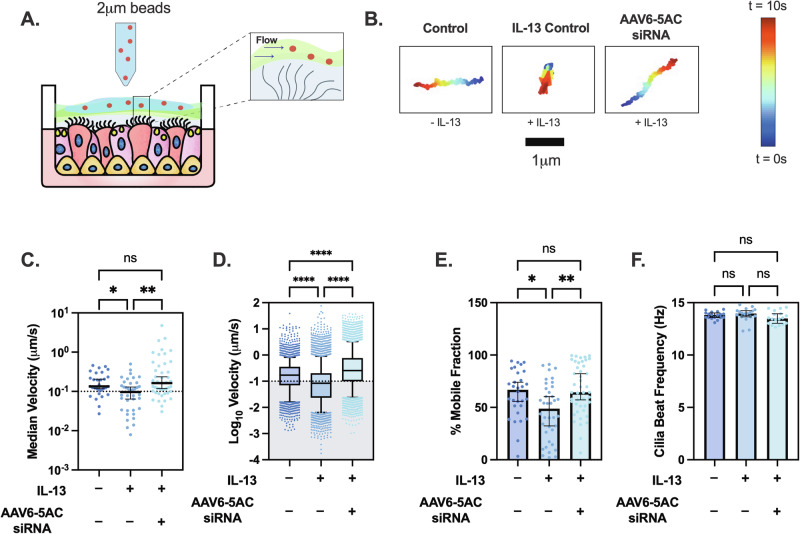
Mucociliary transport is maintained in MUC5AC siRNA treated HAE cultures challenged with IL-13. **A** Schematic showing the design of MCT measurement. Briefly, 2 µm beads are applied onto mucus on ALI cultures. The coordinated beating of cilia causes the mucus with beads to flow, which is imaged. **B** Representative trajectories of single beads in untreated control, IL-13 treated control, and AAV6-5AC siRNA treated group. Dark blue represents trajectories at 0 s and dark red represents trajectories at 10 s. **C** Scatter plot represents median velocities (µm/s) of beads in each video for untreated control, IL-13 treated control, and AAV6-5AC siRNA groups (*n* = 30). **p < 0.01 by Kruskal–Wallis test with Dunn’s correction. **D** Box and Whisker plot representing velocities (µm/s) of all beads in untreated control, IL-13 treated control and AAV6-5AC siRNA groups. Shaded region indicates particles with velocities <0.1 µm/s. ****p < 0.0001 by Kruskal–Wallis test with Dunn’s correction. **E** Scatter plot showing immobile fraction (no. of beads with velocity <0.1 µm/s/ total beads) in each video for untreated control, IL-13 treated control and AAV6-5AC siRNA groups. ***p < 0.001 by Kruskal–Wallis test with Dunn’s correction. **F** Bar graph showing ciliary beat frequency (Hz) in untreated control, IL-13 treated control, and AAV6-5AC siRNA groups.

To evaluate MUC5AC as a suitable target to improve airway clearance in asthma, we measured mucociliary transport rates following treatment with AAV6 expressing MUC5AC siRNA in IL-13 stimulated HAE cultures. Seven days after IL-13 challenge and/or AAV6-5AC siRNA treatment (Fig. 4A), mucociliary transport rate was measured by adding 2 µm red fluorescent beads to the apical surface of transwells and the movement of the beads were tracked in real time using live cell video microscopy (Fig. 5A). We found that beads in IL-13 treated controls traversed less distance compared to beads on untreated controls and AAV6-5AC siRNA treated groups, which were comparable, as seen in representative trajectories (Fig. 5B). We observed that the median transport rates of the beads in each video were comparable between untreated controls and AAV6-5AC siRNA treated groups and significantly reduced in IL-13 treated controls (Fig. 5C). We next wanted to determine how many beads in each video were mobile. We defined beads to be mobile if they traversed a distance greater than or equal to their radii (1 µm) in 10 seconds equivalent to a velocity of 0.1 µm/s. Based on this, beads with speeds less than 0.1 µm/s were considered immobile or stuck (gray shaded region in Fig. 5D). The percentage of mobile particle tracers in each video was then calculated using a 0.1 µm/s velocity cutoff. We found the untreated controls and AAV6-5AC siRNA treated groups had comparable mobile beads ~65% while IL-13 treated controls had significantly fewer mobile beads, less than 50% were mobile (Fig. 5E). To confirm that these changes in transport rates were due to change in secreted mucin composition and not ciliated epithelium function, we measured the ciliary beat frequency which was comparable in all groups (Fig. 5F).

## Discussion

In these studies, we targeted the mucin glycoprotein MUC5AC using AAV vectors to explore its potential as an inhaled gene therapy for asthma. We first assessed the capabilities of AAV6 to successfully deliver cargo into mouse lungs and airways via intranasal administration and determine if relevant epithelial subsets were transduced (e.g. mucus-secreting cells). Previous studies have shown successful AAV6 transduction of airway epithelial cells [27–29], but to the best of our knowledge, transduction in epithelial subsets have not been analyzed. We saw that AAV6 successfully transduced mucus secreting airway epithelial cells in the trachea. Overall, transduction in the lungs was minimal, which is to be expected for the intranasal route of administration. We anticipate higher transduction in the deeper lung via intratracheal modes of administration [31] which will be tested in subsequent in vivo efficacy studies. Detection of AAV6 transduction in goblet cells in the trachea is promising and provides further rationale for using AAV6 to deliver siRNA targeting MUC5AC in asthma. Improved AAV6 variants like AAV6.2FF have shown higher lung transduction efficiency in vivo compared to AAV6 [39–41]. In the future, we aim to test other AAV6 variants with enhanced transduction potential.

A significant biological barrier to inhaled gene delivery vectors is the pathological mucus produced in diseases like asthma. We found in prior work that AAV6 can diffuse through pathological mucus from individuals with CF and transduce airway epithelial cells in vivo [29]. Similarly, we saw that AAV6 was diffusive in pathological in vitro asthma-like (i.e., IL-13 stimulated) mucus, normal mucus, and MUC5B KO (i.e., MUC5AC-rich) mucus collected from ALI cultures. AAV6 diffusion was lower than muco-inert nanoparticles which may possibly be due to interactions of AAV6 capsid proteins with heparan sulfate terminated glycans present in airway mucus [42, 43]. However, overall, AAV6 retained the capacity to transduce the underlying epithelium in IL-13 stimulated, MUC5AC-secreting HAE cultures.

Pathological viscous asthma mucus is the consequence of increased goblet cell number and therefore increased MUC5AC expression [21, 44]. Consistent with prior studies, we observed increased mRNA and protein expression of MUC5AC in IL-13 treated cultures. Pre-treatment with AAV6 expressing MUC5AC siRNA was able to protect cultures when challenged with IL-13, with MUC5AC expression comparable to untreated controls. This was also reflected in the quantification of total O-linked glycosylation in mucus collected from each group. In terms of MUC5B expression, prior literature is inconsistent, with some studies reporting increased MUC5B expression in asthma [45], and others reporting no change [46]. In the current work, we observed no significant differences in MUC5B protein expression between the groups and as such, MUC5AC siRNA treatment did not appear to cause any compensatory expression of MUC5B.

Impaired mucociliary clearance in asthma contributes to obstruction in the airway and creates a niche for exacerbation-initiating infections [19, 47]. As noted, asphyxiation from complete blocking of the airway by intraluminal mucus plugs is the primary cause of death in asthma [48–50]. To determine the potential of the approach to restore normal MCC in asthma, we also evaluated if MUC5AC siRNA delivery with AAV6 would lead to improved MCC in IL-13 stimulated HAE cultures. In IL-13 treated HAE cultures pre-treated with AAV6-5AC siRNA, MCC remained effective despite being challenged with IL-13 with comparable transport velocities and mobile beads to untreated control. These findings suggest a prophylactic strategy to suppress MUC5AC hypersecretion could be useful in preventing mucostasis and generation of airway mucus plugs in asthma.

The present study demonstrated that AAV6 is successfully able to penetrate an asthma-like mucus barrier and transduce IL-13 stimulated HAE cells differentiated at ALI in vitro. We also showed using an in vitro model of IL-13 induced asthma that AAV6 mediated siRNA delivery could effectively prevent MUC5AC hypersecretion and mucociliary dysfunction. For our MUC5AC siRNA treatment studies, we unfortunately were unable to use a higher MOI of 10^5^ as shown to be effective with AAV6-eGFP due to a relatively lower stock titer of AAV6-5AC siRNA. Considering this, we opted to use a two-dose regimen of AAV6-5AC siRNA which proved to be adequate for transduction and expression of MUC5AC siRNA. These studies provide the basis for our future plans to evaluate the effectiveness of AAV6 mediated MUC5AC siRNA treatment in primary HAE cultures from asthmatic donors as well as an in vivo model of allergic asthma. A limitation of the current work is this approach was only tested as a prophylactic treatment prior to establishment of inflammation and mucostasis with IL-13 stimulation. In future work, we plan to assess if MUC5AC siRNA delivery has the potential to reverse MUC5AC hypersecretion in vitro and in vivo where we anticipate higher doses may be necessary. Our study also provides rationale for development of mucin-targeted gene therapies for asthma and potentially other chronic respiratory diseases using AAV6 given its capability to transduce secretory cells in vivo. Overall, our results establish AAV gene therapy for MUC5AC-targeting siRNA delivery as an attractive option to prevent mucus obstruction as a treatment for asthma.

## Materials and methods

### Cell culture

The BCi-NS1.1 immortalized human basal cell line was generously provided by Ronald Crystal (Weill Cornell Medical College) and cultured based on previous methods [36, 51]. MUC5B KO BCi-NS1.1 cultures were generated as previously reported [36] and cultures were prepared from frozen stocks for experiments. BCi-NS1.1 cells were cultured at 37 °C, 5% CO_2_ in a flask with PneumaCult-Ex Plus expansion media (STEMCELL Technologies). At about 80% confluency the cells were detached using 0.05% Trypsin-EDTA for 5 min and seeded on 12 mm rat-tail collagen type-I coated transwell inserts (StemCell Technologies) at 10,000 cells/cm^2^ in Ex Plus medium supplied on both the apical and basolateral compartments, until a confluent monolayer was established. Once confluent, medium from the apical compartment was removed and PneumaCult-ALI media (STEMCELL Technologies) was supplied only to the basolateral compartment to transition the cultures into Air-Liquid Interface cultures. The cells were allowed to differentiate into a pseudostratified mucociliary epithelium for 4 weeks with media changed every other day. To establish an in vitro asthma model, ALI cultures were treated with recombinant human IL-13 (10 ng/ml, Peprotech) added in the basolateral compartment for 7 days.

### Mucus collection

Mucus was collected from fully differentiated ALI cultures once or twice a week. PBS was added to the apical surface of cultures for 30 mins at 37 °C. After 30 min, mucus washings were concentrated by passing through 100 kDa amicon filter and were stored at −80 °C for long-term storage prior to usage.

### Particle Tracking Microrheology (PTM)

Based on previously reported methods [52], carboxylate-modified polystyrene nanoparticles (ThermoFisher) were surface modified with a 5-kDa polyethylene glycol-amine (PEG) coating to ensure that these particles are non-adhesive to mucus. Size and zeta potential of the particles were measured using NanoBrook Omni (Brookhaven Instruments). The nanoparticles had a hydrodynamic diameter of 27.74 ± 10.99 nm. Twenty microliters of mucus was added to the microscopy chamber made from vacuum-grease coated O-rings in which 1 µl of nanoparticles and fluorescently labeled AAV6 respectively (~0.002% w/v) were added and allowed to incubate for 30 min at room temperature. The diffusion of nanoparticles and AAV6 was measured using fluorescence video imaging using Zeiss 800 LSM Microscope at 63x-water immersion objective. Briefly, videos were taken for 10 s at 300 ms exposure time (30 Hz) and analyzed using previously developed MATLAB algorithm [53]. The mean squared displacement (MSD) for each particle for lag time (τ) was calculated as 〈MSD(τ)〉 = 〈[*x*(*t* + τ) – *x*(*t*)]^2^ + [*y*(*t* + τ) – y(*t*)]^2^〉. Five videos were taken for each sample (n$\ge$5) from 5 different fields of view, assessing hundreds of particles combined.

### AAV6-eGFP transduction in vitro

Undifferentiated BCi-NS1.1 cultures were seeded in 6 well plates. At 60-65% confluency, the cells were infected with AAV6 encoding GFP (AAV6-CAG-eGFP, 10^13^ viral particles/ml, AAVner Gene) at multiplicities of infection (MOI) of 1000, 10,000, and 100,000 viral particles per cell. The cells were imaged on a Zeiss 800 LSM Microscope at 72 h post infection and analyzed for GFP expression. The MOI for differentiated cultures was calculated based on initial seeding density on transwells. Differentiated BCi-NS1.1 cultures were transduced with AAV6-eGFP at MOI 100,000 apically in regular and IL-13 stimulated cultures, and the cells were fixed and imaged for GFP expression after 72 h.

### AAV6 MUC5AC siRNA transduction in vitro

AAV6 packaged with MUC5AC siRNA (NM_001304359) was obtained from Applied Biological Materials, Canada. Briefly, ALI cultures were pre-treated apically with 2500 MOI on with their 1^st^ dose on day 15 of differentiation and a second dose on day 18 of differentiation. The IL-13 treated controls were treated with the same volume of PBS. All cultures were washed with PBS on day 21 to remove any untransduced AAV6 from the apical surface. These cultures were challenged with IL-13 (10 ng/ml) supplemented to the media in the basolateral chamber from day 21 to 28 and replenished with every media change. On day 28, the cells were analyzed using PCR, immunofluorescent staining and particle velocimetry measurements of MCC.

### Immunofluorescence

Differentiated and undifferentiated BCi-NS1.1 cells were fixed with 100% ice-cold methanol for 15 mins at 4 °C. The cells were washed with PBS and blocked with 5% bovine serum albumin (BSA) in 0.01% PBST for 1 h at room temperature. For MUC5B (rabbit anti-human, Cell Signaling Technologies, MA) and MUC5AC (mouse anti-human, ThermoFisher Scientific) expression, the cultures were incubated with primary antibodies (1:1000) overnight at 4 °C. The next day, cultures were washed with PBS twice. The cultures were incubated with secondary antibodies AlexaFlour 647 (anti-rabbit, 1:2000) and AlexaFlour 555 (anti-mouse, 1:2000) in PBS for 1 h at room temperature. The cultures were then washed with PBS twice. For cultures with AAV6-eGFP transduction, cultures were fixed, incubated in 1 µg/ml DAPI in PBS for 15 min at room temperature, washed twice with PBS, and then mounted and imaged using Zeiss 800 LSM Microscope. Quantification of integrated density of MUC5B and MUC5AC expression was measured in FIJI image software using the automated thresholding function.

### qRT-PCR

RNA was extracted from differentiated BCi-NS1.1 cultures using the RNeasy minikit (Qiagen) based on manufacturer’s protocol. cDNA from the RNA was prepared with SuperScript III (Invitrogen) as per manufacturer’s protocol. Quantitative PCR for MUC5AC was carried out using PowerUP SYBR Green Master Mix (Applied Biosystems) using forward and reverse primers (Integrated DNA Technologies).

### Mucociliary transport and ciliary beat frequency

Prior to MCC and CBF analyses, cultures were washed, and mucus was allowed to accumulate for 24 h. A 25 µl suspension of 2 µm red-fluorescent polystyrene microspheres (Invitrogen, 1:2000 dilution in PBS) was applied apically to the cultures and allowed to equilibrate at 37 °C for 15 min. Videos of 5 regions were recorded using the Zeiss 800 LSM Microscope at 10× at frame rate of 10 Hz for 10 s. The microsphere tracking analysis is based on a custom MATLAB algorithm^22^. The software computes the trajectories of the fluorescent microsphere in the xy-plane in each frame. The displacement is calculated based on trajectories and velocity is calculated based on displacement over time elapsed. To measure ciliary beat frequency, 20 s videos at frame rate of 50 Hz were recorded at 10× magnification using the Zeiss 800 LSM Microscope from 3 random regions using brightfield. The local pixel intensity maxima were counted using custom written MATLAB algorithm, which indicates beating cilia. The beat frequency was calculated by dividing the number of beats over time elapsed.

### AAV6-mCherry transduction in vivo

Six-week-old male and female BALB/c mice (10 mice total, 5 males and 5 females) were purchased from Charles River Laboratories (Wilmington, MA) and maintained in disease free conditions at the animal facility. The animal study protocol was approved by the Institutional Animal Care and Use Committee (IACUC) of the University of Maryland College Park (protocol R-MAY-22-25). AAV6 encoding mCherry (AAV6-CMV-mCherry, stock: 10^13^ viral particles/ml, AAVner Gene) was intranasally administered to anesthetized mice at a dose of 10^11^ viral particles/mouse. The mice were monitored for 2 weeks and were sacrificed on day 14. Their lungs and trachea were isolated for imaging and flow cytometry analysis as described below.

### Flow cytometry

The lungs and tracheas of mice (*n* = 3 mice per group with 1 male and 2 female) were removed and dissected on petri plates. Single cell suspension of the tissues was made by digesting tissue with Collagenase IV (1 mg/ml, ThermoFisher Scientific), and DNAse I (25 ug/ml, Millipore Sigma) for 45 min at 37 °C. Dispase II (2 mg/ml, Millipore Sigma) was also added for trachea. The tissue was then repeatedly passed through a 70 µm cell strainer until a single cell suspension was obtained. The cells were washed in PBS (300 × *g*, 5 min) twice. The lung cells were suspended in RBC Lysis buffer for 3 min on ice and washed with PBS. The cells were distributed into groups in FACS staining buffer and were stained for different airway epithelial cell markers as shown in Table 1 and flow cytometry was performed (BD FACS Celesta).

**Table 1:** Antibodies used for flow cytometry.

Cell marker	Primary/conjugated antibody	Secondary antibody	Manufacturer
Basal cells (CD271)	AlexaFluor 488 conjugated		eBioscience (#53-9400-42)
Ciliated cells (α-Tubulin)	AlexaFluor 488 conjugated	–	Biolegend (#627906)
Goblet cells (MUC5AC)	Mouse anti-MUC5AC	Anti-mouse AlexaFluor488	ThermoFisher, Biolegend (MA5-12178, 406001)
Secretory cells (TSPAN8)	AlexaFluor 488 conjugated	–	FisherScientific (FAB6524G100)

### Ex vivo cryosectioning and staining

To prepare lungs and trachea for immunofluorescence (*n* = 3 mice per group with 2 male and 1 female), the lungs were inflated with 10% formalin which was delivered through blunt-end needle inserted via the trachea. After inflation, the lungs and trachea were collected and placed in 10% formalin overnight at room temperature. The tissues were transferred to cryomolds with OCT and frozen at −80 °C. Using the Leica CM1950 Cryostat, 10 µm lung sections and 7 µm tracheal sections were made. The sections were allowed to calibrate at room temperature overnight and fixed with 100% methanol and stained with DAPI as per immunofluorescence protocol and imaged at 10× magnification using the Zeiss 800 LSM Microscope.

### Statistical analysis

The data were statistically analyzed using GraphPad Prism Software. Comparison between 2 groups were performed using 2-tailed Student’s *t* test or Mann–Whitney *U* test. For comparison between groups, either one-way analysis of variance (ANOVA) followed by a Tukey post hoc correction was used or Kruskal–Wallis with Dunn’s correction was used for non-Gaussian distributions. All graphs show median values and 5th up to 95th percentiles of data and outliers are included. Differences were considered statistically significant at *p* value < 0.05.

## Supplementary information

Supplemental Materials

## Data Availability

All data are available in the main text or the supplementary materials.

## Code availability

The particle tracking code used in the current work is available at http://site.physics.georgetown.edu/matlab/.
