# Supplementary material for: AAV-mediated MUC5AC siRNA delivery to prevent mucociliary dysfunction in asthma
Source: Gene Ther. 2025 Aug 23;32(5):508–16. doi: 10.1038/s41434-025-00564-3 (PMC12518123; doi:10.1038/s41434-025-00564-3)
Supplement: Supplementary file 1 — Supplemental Materials [file 41434_2025_564_MOESM1_ESM.docx]

**AAV-mediated MUC5AC siRNA delivery to prevent mucociliary dysfunction in asthma**

**Authors**

Sahana Kumar^1^, Maria Corkran^2^, Yahya Cheema^1^, Margaret A. Scull^2*^, Gregg A. Duncan^1*^

**Affiliations**

^1^Fischell Department of Bioengineering, University of Maryland, College Park, MD 20742.

^2^Department of Cell Biology & Molecular Genetics, Maryland Pathogen Research Institute (MPRI) University of Maryland, College Park, MD 20742.

Corresponding authors e-mail: [gaduncan@umd.edu](mailto:gaduncan@umd.edu)

**Supplementary Information**

**
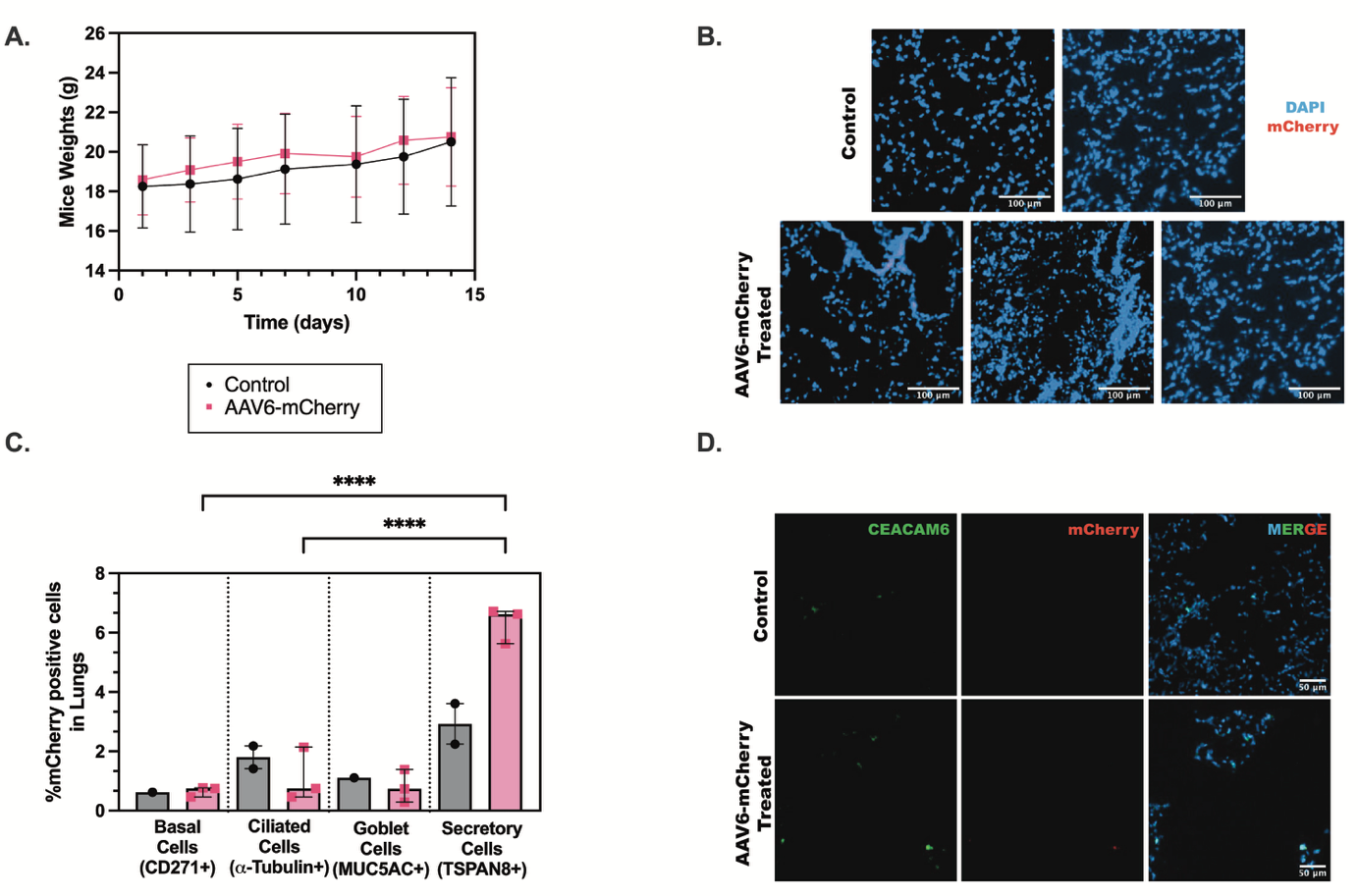
**

**Figure S1: *In vivo* AAV6 transduction in mouse lungs. (A)** Graph showing mouse weights which were recorded every other day throughout the study and showed a steady increase in both control and treated groups. **(B)** Immunofluorescence images of lung sections in control (PBS) and AAV6-mCherry infected mice. mCherry transduction is shown in pink. Scale bar = 100 µm. **(C)** Bar Graphs show % mCherry positive cells normalized to control in different airway epithelial cells from single cell suspension of excised lungs. *****p* < 0.0001 by Ordinary one-way ANOVA. Each dot represents a mouse. **(D)** Immunofluorescence images of lung sections in control (PBS) and AAV6-mCherry infected mice with secretory cell marker Ceacam6 (green). Scale bar = 50 µm

**
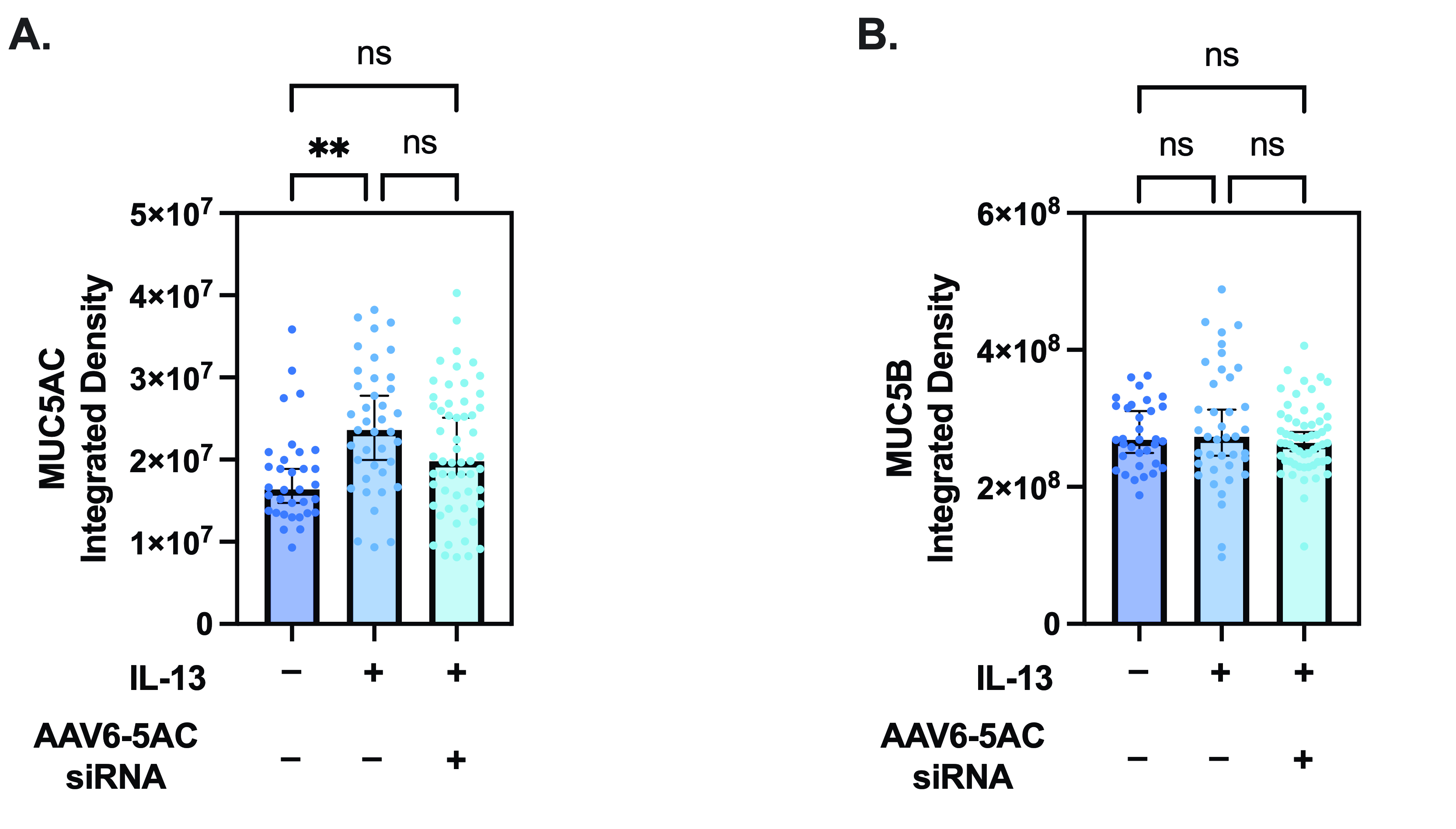
**

**Figure S2: Semi-quantitative comparison of MUC5B and MUC5AC expression in IL-13 stimulated cultures following AAV6-5AC siRNA treatment. (A-B)** Bar graphs showing MUC5AC and MUC5B integrated density respectively, measured from immunofluorescence images (**Figure 4C**) (n≥15, across 9-15 cultures) in untreated controls, IL-13 treated controls, and AAV6-5AC siRNA treated groups. ***p* < 0.001 by One-way ANOVA.
